# The Upregulation of *Cathepsin G* Is Associated with Resistance to Bovine Paratuberculosis

**DOI:** 10.3390/ani12213038

**Published:** 2022-11-04

**Authors:** Maria Canive, Gerard Badia-Bringué, Marta Alonso-Hearn

**Affiliations:** 1 NEIKER-Basque Research and Technology Alliance (BRTA), 20850 Derio, Spain; 2 Doctoral Program in Molecular Biology and Biomedicine, Universidad del País Vasco/Euskal Herriko Unibertsitatea (UPV/EHU), 48940 Leioa, Spain

**Keywords:** paratuberculosis, *Cathepsin G*, monocyte-derived macrophages, mycobacterial growth-inhibition assay, modulation of immune function, polymorphisms, functional variants, cis-eQTL, therapeutics, immunomodulators

## Abstract

**Simple Summary:**

*Cathepsin G* (CTSG) is a serine protease that participates in the killing of pathogens and tissue remodeling at sites of inflammation. We previously found that the presence of the minor allele in the cis-eQTLs-rs41976219 (AC) was associated with increased CTSG mRNA levels in blood samples from Holstein cows. In this study, we test whether genetic variation in the cis-eQTL-rs41976219 is associated with changes in CTSG protein expression and control of *Mycobacterium avium* subsp. *paratuberculosis* (MAP) infection. We demonstrate that the heterozygous genotype for the rs41976219 (AC) results in higher CTSG protein levels in the supernatants of infected CD14+-monocyte-derived macrophages (MDMs) after 2 h of infection and a significantly lower intracellular MAP load at 7 d p.i. than in MDMs from cattle with the AA genotype. Furthermore, the rs41976219 genotype CC is more frequent in healthy cows than in cows with PTB-associated lesions in gut tissues. Higher CTSG levels are detected in plasmas from cows with the CC genotype when compared with cows with the AA + AC genotypes for the rs41976219. We conclude that the presence of the minor allele in the rs41976219 increases the *CTSG* expression in plasma samples and MAP-infected macrophages, and can influence PTB pathogenesis by modulating MAP load within infected macrophages.

**Abstract:**

An in silico genomic–transcriptomic combined approach allowed the identification of a polymorphism (cis-eQTL-rs41976219) in the *Bos taurus* genome associated with the CTSG mRNA expression in bovine blood samples, which suggests that individual genetic variation might modulate the *CTSG* transcriptional response. In the current study, a sandwich ELISA is used to measure the CTSG protein levels in supernatants of monocyte-derived macrophages (MDMs) isolated from cows with the AA (*n* = 5) and AC (*n* = 11) genotypes for the rs41976219 and infected ex vivo with MAP. Cows with the AC genotype have significantly higher CTSG protein levels (1.85 ng/mL) in the supernatants of enriched CD14+-MDMs after 2 h of infection when compared with infected CD14+-MDMs from cows with the AA genotype (1.68 ng/mL). Statistically significant differences in the intracellular MAP load at 7 d p.i. are observed between animals with the AA (2.16 log CFUs) and AC (1.44 log CFUs) genotypes. Finally, the association between the rs41976219 allelic variants and resistance to PTB is tested in a larger cattle population (*n* = 943) classified according to the presence (*n* = 442) or absence (*n* = 501) of PTB-associated lesions. The presence of the two minor alleles in the rs41976219 (CC) is more frequent among healthy cows than in cows with PTB-associated lesions in gut tissues (2.2% vs. 1.4%, OR = 0.61). In agreement with this, the CTSG levels in plasma samples of cows without lesions in gut tissues and with the CC (*n* = 8) genotype are significantly higher than in the plasmas of cows with the AA + AC (*n* = 36) genotypes.

## 1. Introduction

Paratuberculosis (PTB) is a chronic granulomatous enteritis of ruminants caused by *Mycobacterium avium* susbp. *paratuberculosis* (MAP). PTB is a major problem for animal health and the dairy industry. Therefore, the World Organization for Animal Health (WOAH) requires member countries to maintain epidemiological surveillance with the notification of disease cases. The economic impact of PTB on the US dairy industry has been estimated between USD 250 million per year to USD 1.5 billion annually [1]. In Europe, it has been estimated at USD 364.31 million per year. According to their extension in the intestine, cellular infiltrate, and amount of MAP, PTB-associated lesions were classified into focal, multifocal, and diffuse (diffuse paucibacillary or lymphoplasmacytic, diffuse intermediate, and diffuse multibacillary or histiocytic) [2,3]. In addition, MAP has been related to human gastrointestinal inflammatory diseases, such as Crohn’s disease (CD), inflammatory bowel disease (IBD), ulcerative colitis, and IBD-associated colorectal cancer [4,5]. MAP has also been associated with multiple sclerosis, rheumatoid arthritis, and diabetes type 1 [6,7,8,9]. There is no effective treatment for MAP infection and vaccination with heat-inactivated vaccines interferes with the diagnosis of *Mycobacterium bovis* [10,11,12].

Several studies have demonstrated that host genetics governs variations in susceptibility to MAP infection, and therefore selective breeding could decrease the susceptibility to MAP infection [13]. In recent years, genome-wide association studies (GWAS) have identified single nucleotide polymorphisms (SNPs) associated with susceptibility to MAP infection [14,15,16,17]. However, limited progress has been made in identifying SNPs associated with resistance to MAP infection. Moreover, how the identified genetic variants exert their effect is unknown and only a few functional mutations associated with susceptibility to MAP infection have been identified [18,19]. In 2001, the concept of “genetical genomics” where genomic variants are associated with transcript abundance was introduced by Jansen and Nap [20]. This concept supports the idea that integrating genotypes and gene-expression data could contribute to a better understanding of the susceptibility or resistance to microbial infections. Recently, a combined genomic–transcriptomic approach allowed for the identification of several cis-eQTLs, SNPs associated with gene expression and usually located within 1 Mb upstream of the transcription start site of a gene locus, significantly associated with PTB susceptibility [21]. More specifically, 192 cis-eQTLs were significantly associated (FDR ≤ 0.05) with the expression of 145 genes in peripheral blood (PB) samples obtained from Holstein cows with different types of PTB-associated lesions or without lesions in gut tissues [21]. One cis-eQTL, the rs41976219, located on Bos Taurus chromosome 21, was associated with the *Cathepsin G* (CTSG) mRNA expression in PB samples. The minor allele in the cis-eQTLs-rs41976219 (AC) was associated with higher CTSG mRNA expression levels (mean = 114.75 fragments per kilobase per million, FPKMs) while the most frequent homozygous genotype (AA) correlated with very low levels of CTSG mRNA (mean = 0.23 FPKMs).

Neutrophil and macrophage function depends on, among other factors, the presence of *Cathepsins* (*CTS*) that are involved in the innate (pathogen-recognizing and killing) and adaptive (antigen-processing and presentation) responses facilitating rapid pathogen elimination and the induction of long-term immunity [22]. The name “*Cathepsin*” refers to two serine proteases (*CTS A* and *G*), two aspartic proteases (*CTS D* and *E*), and eleven lysosomal cysteine proteases (*CTS B*, *C*, *F*, *H*, *K*, *L*, *O*, *S*, *V*, *X*, and *W*) [23,24]. Following synthesis, *CTSs* are transported to the endosomal/lysosomal compartment where they are activated following the removal of the N-terminal propeptide [25,26]. The presence of *CTSs* in the endo-lysosomal compartment of infected monocytes directs the interaction with and killing of bacteria and contributes to the processing of bacterial antigens for the induction of adaptive immune response [27]. It is not surprising that bacteria manipulate the expression of *CTSs* to favor their intracellular survival in monocytes. On the other hand, we hypothesize that the presence of SNPs in the host genome might also influence *CTSs’* expression and subsequent proteolytic activity. Understanding how the individual regulation of *CTS* expression participates in the pathogenesis of mycobacterial infections is needed for the development of novel therapeutical alternatives to antibiotics. In this study, we test whether individual genetic variation in the cis-eQTL-rs41976219 is associated with changes in CTSG protein expression, the control of MAP infection, and PTB progression.

## 2. Materials and Methods

### 2.1. Animals

For the mycobacterial growth-inhibition assays (MGIAs) and the CTSG quantification assay, PB samples were collected from 16 Holstein Friesian cows from a commercial dairy farm in the Basque Country (Spain). Only adult cows (2 years or older)were included in the study. Animals were not diagnosed with any disease in the period during which samples were collected, and all of them were ELISA negative for the detection of anti-MAP antibodies in the serum at the sampling time (IDEXX Map Ab test, Montpellier, France).

The case–control population consisted of 940 culled Holstein cattle from eight Spanish regions: Basque Country (*n* = 401, 42.52%), Catalonia (*n* = 211, 22.38%), Navarre (*n* = 193, 20.47%), Cantabria (*n* = 65, 6.89%), Aragon (*n* = 35, 3.71%), Castile and Leon (*n* = 24, 2.55%), La Rioja (*n* = 7, 0.71%), and Asturias (*n* = 4, 0.41%). Three slaughtered cows of unknown origin were also included in the study. Only cows (2 years or older, 5.6 years mean age) were included in the analysis. The infection status of the 943 culled animals was determined by the histopathological analysis of gut tissues, as previously described [28]. The stained tissue sections were examined by light microscopy and classified into four groups: those with no lesions, and those with focal, multifocal, or diffuse lesions [2].

### 2.2. MAP Strain, Bacterial Culture, and Preparation of Bacterial Suspensions

MAP reference strain K10 was obtained from the American Type Culture Collection (ATCC) (Manassas, VA, USA). The bovine K10 isolate of MAP was grown in T25 tissue culture flasks at 37 ± 1 °C in 8 mL of Middlebrook 7H9 broth (Difco Laboratories, Detroit, MI, USA) supplemented with 10% (*v*/*v*) oleic acid-albumin-dextrose-catalase (Becton, Dickinson and Company, Franklin Lakes, NJ, USA), 0.05% (*v*/*v*) Tween-80 (Sigma-Aldrich, St. Louis, MO, USA), and 2 mg/L of mycobactin J (Allied Monitor Inc., Fayette, MO, USA) for 20 days at 37 °C. Bacterial cells were harvested by centrifugation at 3000× *g* for 20 min in a Beckman Coulter Allegra X-12 centrifuge. Then, bacterial pellets were resuspended in 2 mL of Hank’s balanced salt solution (HBSS), and the resultant suspension was passed 20 times through a 27-gauge needle. The turbidity of the bacterial suspension was adjusted to a McFarland standard of 1 with a Densimat (bioMerieux, Marcy l’Etoile, France). Only the top fraction of the suspension containing dispersed bacteria was used for the infection assays.

### 2.3. Peripheral Blood Mononuclear Cells’ (PBMCs) Purification, CD14+ Monocyte Selection, Ex Vivo Differentiation to Monocyte-Derived Macrophages (MDMs), and MAP Infection

PBMCs were isolated from PB using Ficoll-Paque density gradient centrifugation. Briefly, fifteen milliliters of PB were drawn from the tail vein of healthy Holstein cows into heparinized Vacutainer tubes (Becton, Dickinson and Company, Sparks, MD, USA) and diluted at 1:2 in Hanks balanced salt solution (HBSS). Leucosep tubes (Greiner, Kremsmünster, Austria) were filled with 15 mL of Ficoll-Paque gradient (1.084 g/cm^3^) (GE HelthCare, Uppsala, Sweden) and centrifuged at 1000× *g* for 30 s at room temperature. Subsequently, the diluted blood was overlaid on the top of the Ficoll-Paque and centrifuged at 800× *g* for 15 min. The plasma layer was removed and the cell interphase containing PBMCs was collected and transferred to a clean tube. PBMCs were washed twice in HBSS and centrifuged at 400 g for 10 min to remove platelets. Additionally, CD14+ monocytes were magnetically enriched from PBMCs using CD14+ microbeads and magnetically activated cell-sorting (MACS) technology (Miltenyi Biotech, Bergisch Galdbach, Germany), as previously described [29]. CD14+ monocytes were enriched from PBMCs to maximize monocyte purification and subsequent differentiation to MDMs. PBMCs and CD14+-selected monocytes were resuspended in RPMI-1640 supplemented with 20 mM L-glutamine, 10% heat-inactivated bovine serum, 100 U ml-1 penicillin G, and 100 mg ml-1 streptomycin sulfate (Lonza, Spain), and seeded into 24-well plates at a density of 1 × 10^6^ cells/mL. The cells were incubated overnight at 37 °C in a humidified 5% CO_2_ incubator. The following day, non-adherent cells were washed with PBS, and adherent cells were incubated in medium for 7 days at 37 °C to allow for differentiation into MDMs before infection. MDMs and CD14+-MDMs were inoculated in duplicate with a single-cell suspension of MAP K10 strain at a multiplicity of infection (MOI) of 10:1 (bacteria:cell). At 2 h p.i., the supernatants were removed and the cells were washed twice with HBSS to remove extracellular bacteria. The infected MDMs were lysed at this time (2 h p.i.) or cultured at 37 °C for 7 d in medium. At each time point, the infected MDMs were lysed with 0.5 mL of 0.1% Triton X-100 (Sigma-Aldrich) in sterile water for 10 min.

### 2.4. Mycobacterial Growth-Inhibition Assay (MGIA) Assessment Using the BACTEC MGIT 960 System

The MGIA assessment using the BACTEC MGIT 960 system (Becton, Dickinson and Company, Sparks, MD, USA) was performed, as previously described [21,29,30]. Each supplemented mycobacteria growth-indicator tube (MGIT) contained 7 mL of modified Middlebrook 7H9 broth base with casein peptone and an oxygen-sensitive fluorescent compound (tris-4,7-diphenyl-1,10-phenathroline ruthenium chloride pentahydrate) embedded in silicone on the bottom of the tube. The fluorescent compound fluoresced upon exposure to CO_2_ produced from metabolically active mycobacteria. Each tube was supplemented with 800 µL of an enrichment supplement (BBL MGIT OADC growth supplement) and an antibiotic mixture (BBL MGIT PANTA Antibiotic Mixture) (Becton, Dickinson, and Company). The tubes were also supplemented with 2 µg ml^−^^1^ of mycobactin. The MGIT tubes were inoculated with 0.1 mL of the initial MAP suspension and with 0.5 mL of the 2 h and 7 d p.i. cell lysates. The tubes were incubated at 37 ± 2 °C for up to 42 days in a Bactec MGIT 960 instrument (Becton, Dickinson, and Company). The earliest instrumental indicator of positivity (time to detection (TTD)) for each tube was recorded. The predicted number of bacteria in each positive tube was calculated using standard curves that related TTD to the estimated log CFUs [30]. For the standard curves, a 10-fold dilution series of the MAP cellular suspension (McFarland = 1) was prepared and the TTDs of 100 µL of each dilution in the BACTEC MGIT 960 system were recorded. MAP (log CFUs) was plotted versus TTDs (in days) and the obtained mathematical equation was used to determine the estimated log CFUs for each sample. The log CFU ratios were calculated by dividing the estimated log CFUs at day 7 by that at 2 h p.i. Lower log-CFU ratios reflected greater resistance to the infection. A Student’s *t*-test was performed for a comparison of the mean log CFUs between two groups (GraphPad Prism 8, San Diego, CA, USA). Differences were considered significant when *p*-values were ≤0.05.

### 2.5. Bovine CTSG ELISA

The CTSG levels in plasma samples and supernatants of MAP-infected MDMs were assessed by a quantitative sandwich ELISA according to the manufacturer’s instructions (MyBioSource, San Diego, CA, USA). The sensitivity of this kit was 0.1 ng/mL and the detection range was 0.25–8 ng/mL. Statistical analysis was performed using an unpaired *t*-test for the comparison of the mean CTSG concentration between the two groups (GraphPad Prism 8, San Diego, CA, USA). Differences were considered significant when *p*-values were ≤0.05.

### 2.6. Genotyping, Case–Control Population, and Odds Ratio (OR)

The blood samples of all the animals included in the study were collected from the coccygeal (tail) vein of each animal into EDTA Vacutainer tubes (BD Vacutainer System, Becton, Dickinson, and Company, Sparks, MD, USA). Genomic DNA was extracted from the blood buffy coat using the QIAmp DNA Blood Mini Kit, according to the manufacturer’s instructions (Qiagen, Hilden, Germany). Purified genomic DNA was quantified spectrophotometrically and subsequently genotyped with the EuroG10K MD BeadChip at the molecular genetic laboratory service of the Spanish Federation of Holstein Cattle (CONAFE) using the Infinium iScan software for allele assignation (Illumina, San Diego, CA, USA). Genotypes obtained from this case–control population have been used previously for several GWAS studies [16,17,31].

To quantify the strength of the association between the rs41976219 genotypes and the presence or absence of PTB-associated lesions, ORs were calculated using logistic regression analysis under five different genetic models (co-dominant, dominant, recessive, over-dominant, and log-additive) with SNPassoc 2.0-11 (Victor Moreno, Juan R Gonzalez, Dolors Pelegri, Barcelona, Spain). Age was included as a covariate in the analysis. If the OR was less than 1, the presence of one event (a specific genotype) reduced the odds of the other event (presence of PTB-associated lesions). For each genetic model, SNPassoc provided genotype frequencies, ORs, and 95% CI with the major homozygous genotype deemed as the baseline.

## 3. Results

### 3.1. The Presence of the Minor Allele in the rs41976219 (AC) Resulted in Increased CTSG Protein Levels in Supernatants of MAP-Infected CD14+-MDMs after 2 h of Infection

**Figure 1:**
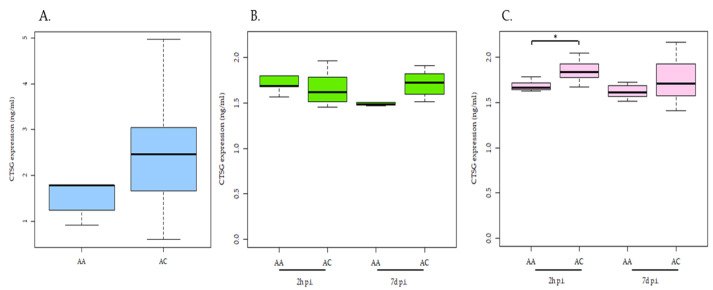
CTSG levels in carriers of different genotypes of cis-eQTL-rs41976219. MDMs and CD14+-MDMs purified from 16 cows with the homozygous (AA; *n* = 5) and heterozygous (AC; *n* = 11) genotypes for rs41976219 infected ex vivo with MAP for 2 h and 7 days. CTSG levels measured in plasmas (**A**) and supernatants of MDMs (**B**) and CD14+-MDMs (**C**) after 2 h and 7 d of MAP infection by ELISA. Statistically significant differences are observed in the mean CTSG levels of expression in the supernatants of MAP-infected CD14+-MDMs after 2 h of infection depending on the genotype of the cis-eQTL-rs41976219. The median and upper and lower quartiles of each group are represented by horizontal and vertical lines, respectively. The lowest point is the minimum of the dataset and the highest point is the maximum of the dataset. *p*-values are calculated using an unpaired *t*-test. * *p*-value ≤ 0.05.

The heterozygous genotype (AC) in the cis-eQTL-rs41976219 was previously associated with higher CTSG mRNA expression (21). Consequently, the rs41976219 genotypes may directly impact CTSG protein expression. To address this, MDMs and CD14+-MDMs purified from 16 cows with the homozygous (AA; *n* = 5) and heterozygous (AC; *n* = 11) genotypes for the rs41976219 were infected ex vivo with MAP for 2 h and 7 days. Using a bovine CTSG quantitative sandwich ELISA, the CTSG protein levels were measured in plasma and supernatants obtained from MAP-infected MDMs and CD14+-MDMs purified from cows with the AA and AC genotypes for the rs41976219. As presented in Figure 1, we can observe that the CTSG levels are significantly higher in the supernatants of MAP-infected CD14+-MDMs obtained from cows carrying the C allele (1.85 ± 0.11 ng/mL) after 2 h of infection than in infected CD14+-MDMs from cows with the AA genotype (1.68 ± 0.06 ng/mL) (*p* = 0.008). No significant differences in the CTSG protein levels in the supernatants of MAP-infected MDMs between cows with the AA and AC genotypes were observed. Although higher CTSG protein levels were also observed in the plasmas of cows with the AC genotype (2.72 ± 1.64 ng/mL) when compared with the AA genotype (1.67 ± 0.65 ng/mL), this difference was not statistically significant (*p* = 0.19). Our results confirm that an increase in CTSG mRNA expression correlates with higher levels of CTSG protein expression in the supernatants of MAP-infected CD14+-MDMs obtained from cows with the AC genotype following 2 h of infection. The use of selected CD14+-MDMs instead of MDMs presented better variation in the genotype-dependent CTSG expression after MAP infection.

### 3.2. Association between the Inhibition of MAP Growth within MDMs and the cis-eQTL-rs41976219 AC Genotype Using an MGIA Assay

**Figure 2:**
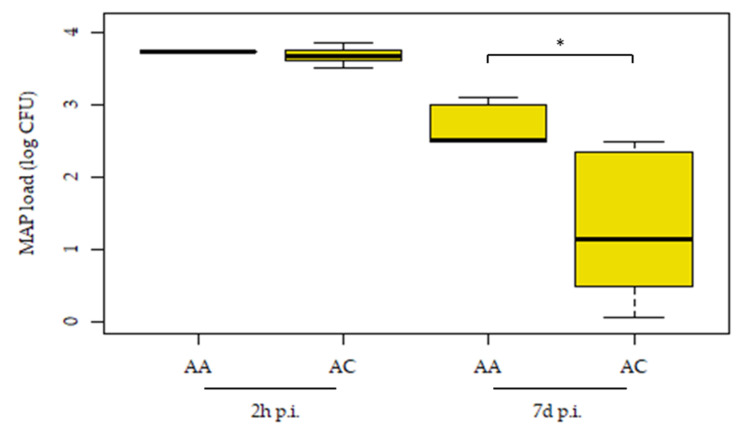
Host MDMs expressing the cis-eQTL-rs41976219 AC genotype show an increased ability to control MAP infection. MDMs purified from PB of cows with the homozygous (AA, *n* = 5) and heterozygous (AC, *n* = 11) genotypes for the rs41976219 and infected ex vivo with MAP. Intracellular bacterial load estimated at 2 h and 7 days p.i. The median and upper and lower quartiles of each group are represented by horizontal and vertical lines, respectively. The lowest point is the minimum of the dataset and the highest point is the maximum of the dataset. *p*-values are calculated using an unpaired t-test. * *p*-value ≤ 0.05.

The association between MDMs’ resistance to MAP infection and the cis-eQTL-rs41976219 genotypes was tested using an ex vivo MGIA assay (Figure 2). MDMs were purified from the PB of cows with homozygous (AA, *n* = 5) and heterozygous (AC, *n* = 11) genotypes for the rs41976219 and infected ex vivo with MAP. The bacterial load was estimated at 2 h and 7 d p.i. using the BACTEC MGIT system. MDMs obtained from cows with the AA genotype presented a greater MAP uptake at 2 h p.i. when compared with cows with the AC genotype; 3.74 ± 0.19 and 3.69 ± 0.17 log CFUs, respectively. Although the presence of the AC genotype reduced MAP internalization within MDMs, this difference was not statistically significant (*p*-value = 0.409). Statistically significant differences in the intracellular MAP load at 7 days p.i. were observed between animals with the AA (2.32 ± 1.06 log CFUs) and AC (1.44 ± 0.97 log CFUs) genotypes (*p* = 0.028). Moreover, statistically significant differences in the ratios (log CFU 7 d/log CFUs 2 h) of the animals with the AA (0.58) and AC (0.33) genotypes were observed (*p* = 0.04). These results suggest a significant effect of the minor allele in the rs41976219 on limiting/controlling intracellular MAP and confirms that cis-regulatory variation modulates resistance to MAP infection.

### 3.3. The cis-eQTL-rs41976219 T/T Genotype Correlated with PTB Control

The infectious status of the animals included in the case–control study (*n* = 943) was previously determined by the histopathological analysis of gut tissues [28]. To quantify the strength of the association between the rs41976219 genotypes and the presence or absence of PTB-associated lesions, ORs were calculated using logistic regression analysis. Table 1 presents the allelic frequencies of the rs41976219 in cases and controls and the calculated ORs. As can be observed with the recessive model, the CC genotype in the cis-eQTL-rs41976219 was more frequent among healthy cows (2.2%) compared to cows with PTB-associated lesions in gut tissues (1.4%); OR = 0.61 (CI: 0.22–1.67).

### 3.4. The Presence of Two Minor Alleles in the cis-eQTL-rs41976219 (CC) Increased CTSG Protein Levels in the Plasmas of Healthy Cows

**Figure 3:**
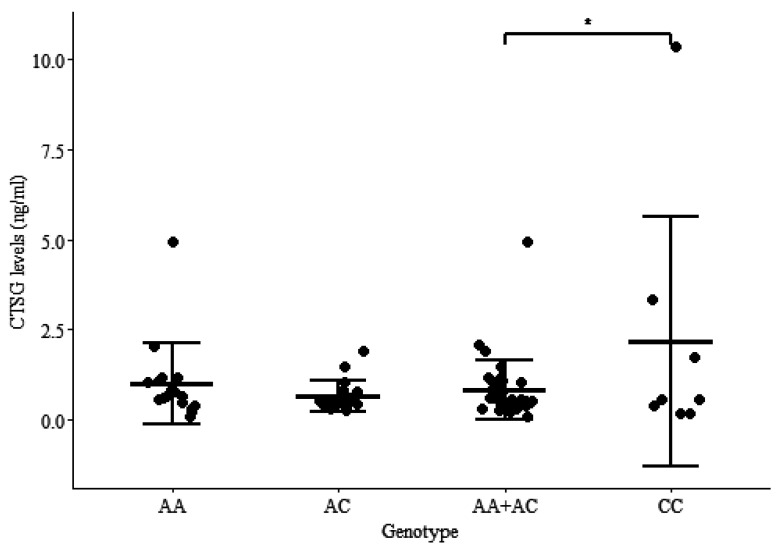
Plasma CTSG levels in carriers of different genotypes of cis-eQTL-rs41976219. CTSG levels measured in the plasmas of cows without lesions in gut tissues and with the AA, AC, and CC genotypes for cis-eQTL-rs41976219 by ELISA. Data are shown as the mean ± SD of 44 cattle, 8 carriers of the CC, 17 of the AA, and 19 of the AC genotypes. Statistically significant differences are observed in the mean CTSG levels in the plasmas of cows with the CC genotype when compared with cows with the AA + AC genotypes. *p*-values are calculated using an unpaired *t*-test. * *p*-value ≤ 0.05.

The CTSG levels were measured by ELISA in plasma samples of cows without lesions in their gut tissues and with the CC (*n* = 8), AA (*n* = 17) and/or AC (*n* = 19) genotypes. As observed in Figure 3 the CTSG levels in plasmas of cows carrying the CC genotype are significantly higher (2.15 ng/mL) than in the plasmas of cows displaying the AA and AC genotypes (0.82 ng/mL) for rs41976219 (*p* = 0.039).

## 4. Discussion

To control MAP infection, we first need to understand the host–pathogen interactions [32,33,34,35]. Second, developing new diagnostic tools or improving the sensitivity of the ones already existing would help diagnose the infection earlier and avoid the late stages of the disease [36,37]. This should be followed by developing new and affordable therapeutic approaches for the treatment of infected animals and the prevention of MAP spread. At present, it is widely accepted that host antimicrobial proteins and peptides able to target the intracellular bacteria without damaging the host play important roles in the mammalian innate defense and often have immunomodulatory effects [38]. Although therapeutic interventions with these immunomodulatory agents could control intracellular pathogens, including MAP, research on host antimicrobial proteins and peptides against MAP is still scarce. A previous study demonstrated that synthetic *Cathelicidin* LL-37 reduces MAP internalization and pro-inflammatory cytokines in macrophages [39].

The CTSG was the first human PBMC protein proven to have antibacterial activity in vitro against Gram+ and Gram− bacteria [40,41,42]. CTSG is known for its ability to kill pathogens and also for its role in tissue remodeling and inflammation resolution. CTSG is a 26 kDa serine protease expressed in azurophilic granules of neutrophils, B cells, myeloid dendritic cells (DCs), plasmacytoid DCs, and cells of the monocyte/macrophage lineage [43,44]. Although CTSG is mainly located in the endo-lysosomal compartment, it is also active in the cytosol, extracellular space, and cell surface. CTSG is released after cell infection enhancing *Platelet-activating Factor*, *Tumor Necrosis Factor α*, *Interleukin 1β*, and *Interleukin-8 (CXCL8/IL8)* production. *CXCL8/IL8* acts as a strong neutrophil chemoattractant to the site of infection and as a major proinflammatory cytokine. Since the presence of CTSG in the endo-lysosomal compartment of infected cells permits direct interaction with and killing of bacteria, it is not surprising that mycobacteria can control CTSG expression and activity to favor their intracellular survival in monocytes [27]. A comparative study using a pathogenic strain *M. tuberculosis* and a non-pathogenic strain *M. smegmatis* revealed that following infection with *M. tuberculosis*, mRNAs for the majority of *CTSs* were downregulated in human primary macrophages (*CTSB*, *C*, *D*, *E*, *G*, *K*, *O*, *S*, *V*, and *W*), in contrast to the non-pathogenic *M. smegmatis* that induced the upregulation of most *CTSs.* Such a global downregulation of *CTSs* expression decreased pathogen killing and improved intracellular bacterial survival [45]. More specifically, the mechanism that *M. tuberculosis* uses to control the expression and proteolytic activity of CTSG was proposed by Danelishvili et al. [46]. They proposed that the bacterial protein Rv3364c, secreted by infected macrophages, binds to and inhibits the activity of membrane CTSG leading apoptosis inhibition. Walter et al. conducted a survival study using mice deficient in *CTSs G*, *K*, *L*, *B*, and *Z* and infected with *M. tuberculosis* [47]. Their results showed that only mice deficient in serine protease CTSG succumbed to *M. tuberculosis* infection more rapidly than wild-type mice, whereas mice deficient in the other *CTSs* did not demonstrate such an effect. In the context of MAP infection, our previous RNA-sequencing analysis revealed the downregulation of *CXCL8/IL8* in the PB and ileocecal valve (ICV) of MAP-infected cattle, regardless of the stage of the infection [35]. In addition, the downregulation of the CTSG mRNA expression was also observed in PB samples of MAP-infected animals suggesting the inhibition of neutrophil recruitment and CTSG-dependent killing response upon MAP infection [35].

Genomic-transcriptomic approaches allow for the identification of SNPs that affect the expression levels of genes associated with complex traits. We previously observed that the minor allele in cis-eQTLs-rs41976219 was associated with individual increased CTSG mRNA levels in blood samples obtained from Holstein cows [21]. In contrast, the CTSG mRNA was practically absent in the blood samples of cattle with the AA genotype. In the current study, we demonstrated that the heterozygous genotype for rs41976219 (AC) resulted in higher CTSG protein levels in the supernatants of infected MDMs after 2 h of infection and a significant lower intracellular MAP load at 7 d p.i. when compared with cattle with the AA genotype. Since it was difficult to find animals with the two minor alleles (CC) for the rs41976219 due to its low frequency, only animals with the genotypes AA and AC were included in the first part of the study. Although the number of animals was low (*n* = 16), we demonstrated that the heterozygous genotype for the rs41976219 (AC) resulted in higher CTSG protein levels in the supernatants of infected CD14+-MDMs following 2 h of infection and significantly lower intracellular MAP load at 7 d p.i. than in MDMs from cattle with the AA genotype. The association between the CC allelic variant and resistance to PTB was subsequently demonstrated in a larger cattle population (*n* = 943) using logistic regression analysis. In addition, the CTSG levels in plasma samples of cows without lesions in gut tissues and with the CC (*n* = 8) genotype were significantly higher than in the plasmas of cows with the AA + AC (*n* = 36) genotypes (*p* = 0.039). Since some of the animals without lesions might not have been exposed to MAP, variability was observed in the levels of CTSG in the plasmas of the animals with the CC genotype. Consequently, more samples are needed to support our data. On the other hand, MAP infection blocks CTSG expression in the blood of infected animals (35). Therefore, plasma samples obtained from animals with lesions were not tested by ELISA.

In agreement with our results, Rivera-Marrero et al. demonstrated that in THP-1 cells, the expression and activity of CTSG were downregulated upon exposure to a pathogenic strain of *M. tuberculosis*, and that the addition of CTSG to THP-1 cells before infection decreased *M. tuberculosis* viability [48]. In the alveolar macrophages of mice infected with the attenuated *M. bovis* bacillus Calmette–Guérin (BCG) strain, CTSG is strongly upregulated and, together with *neutrophil elastase* (*NE*), participates in the effective elimination of pathogens [49]. Although engulfed pathogenic mycobacteria induce the arrest of phagosome maturation and acidification in macrophages, CTSG and *NE* are neutral serine proteases and so can optimally digest the engulfed bacteria at a neutral pH level. Moreover, Steinwede et al. demonstrated that human CTSG encapsulated in liposomes reduced the mycobacterial load in the lungs of mice infected with pathogenic *M. bovis* [50].

## 5. Conclusions

CTSG is relevant for the control of MAP infection, but pathogenic mycobacteria, including MAP, downregulate the levels of CTSG in infected macrophages to facilitate their survival. On the other hand, individual genetic variation affects CTSG expression levels in MAP-infected MDMs. Our study revealed that the CTSG levels were significantly higher in the supernatants of MAP-infected CD14+-MDMs obtained from cows carrying the AC genotype for rs41976219 compared to those detected in supernatants obtained from cows with the most abundant AA genotype. Interestingly, MDMs obtained from hosts with the AC genotype for rs41976219 exhibited an enhanced ability to clear MAP infection. Furthermore, the rs41976219 genotype CC was more frequent in healthy cows than in cows with PTB-associated lesions in their gut tissues. Higher CTSG levels were measured in the plasmas of healthy cows with the CC genotype when compared with cows with the AA and AC genotypes. We can conclude that the presence of the minor allele in ciseQTL-rs41976219 increases CTSG expression in plasma samples and MAP-infected macrophages, and contributes to the control bacterial load within infected macrophages. 

## Figures and Tables

**Table 1:** Frequencies of the rs41976219 genotypes in healthy cows and cows with PTB-associated lesions and odds ratios (ORs) for each genetic model.

	No Lesion (N)	Frequency (%)	Lesion (N)	Frequency (%)	OR (95% CI)
**Codominant**					
AA	422	84.2	369	83.5	1
AC	68	13.6	67	15.2	1.13 (0.78–1.62)
CC	11	2.2	6	1.4	0.62 (0.23–1.7)
**Dominant**					
AA	422	84.2	369	83.5	1
AC-CC	79	15.8	73	16.5	1.06 (0.75–1.5)
**Recessive**					
AA-AC	490	97.8	436	98.6	1
C/C	11	2.2	6	1.4	0.61 (0.22–1.67)
**Overdominant**					
AA-CC	433	86.4	375	84.8	1
AC	68	13.6	67	15.2	1.14 (0.79–1.64)
**log-Additive**					
0, 1, 2	501	53.1	442	46.9	1

## Data Availability

The original contributions presented in this study are included in the article. Further inquiries can be directed to the corresponding authors.
